# Longitudinal Changes in the Government-Certified Index Stage and Requisite Costs for Long-Term Care Insurance System among the Community-Dwelling Demented Elderly in Japan

**DOI:** 10.1155/2013/164919

**Published:** 2013-02-28

**Authors:** Shunichiro Shinagawa, Shiori Nakamura, Makoto Iwamoto, Norifumi Tsuno, Masahiro Shigeta, Kazuhiko Nakayama

**Affiliations:** ^1^Department of Psychiatry, Jikei University School of Medicine, 3-25-8 Nishi-shinbashi, Minato-ku, Tokyo 105-8461, Japan; ^2^Kiyokawa-Enju Hospital, 3414 Kiyokawa, Aiko District, Kanagawa 243-0112, Japan; ^3^Faculty of Health and Sciences, Tokyo Metropolitan University, 7-2-10 Higashiogu, Arakawa-ku, Tokyo 116-8551, Japan

## Abstract

*Background*. A new public long-term care (LTC) insurance was launched in 2000 in Japan. However, there have been few studies involving factors that increase LTC costs of demented subjects; no follow-up studies involving the Government-Certified Index (GCI) and requisite costs related to the causes of dementia. *Method*. An epidemiological survey was conducted in a rural area in Japan in 1999, and 271 subjects were diagnosed as dementia patients. Age, sex, mini-mental state examination, clinical dementia rating, activity of daily living, causes of dementia, and coexisting physical disease were confirmed. After the LTC insurance has been launched, we tracked the GCI stages and payment amounts every month for 8 years. *Result*. 209 subjects were certified to be eligible for LTC insurance; however, 13 did not receive any payment. Only 49 out of 209 were alive after the follow-up period. The most common cause of dementia was Alzheimer's disease (AD), followed by vascular dementia (VaD). There was no significant difference between the mortality rates of the two groups. VaD subjects required higher costs than AD subjects in the total certified period and in GCI stage 5. *Conclusion*. Our results indicate that causes of dementia can have an impact on the requisite costs for the LTC insurance.

## 1. Introduction

A growth of the elderly population consequently increases the number of demented subjects, and dementia is one of the major challenges of health care systems in most countries, especially in aging societies such as Japan. The presence of dementia is strongly related to a decline in the level of daily functioning [1], and the severity of dementia is one of the predictors of higher levels of care [2]. Furthermore, the presence of dementia has an impact on patients' physical condition, increase the burden on caregivers [3], and can even diminish the life expectancy of elderly people [4, 5].

In Japan, the public long-term care (LTC) insurance system was launched in 2000 to deal with an increasing number of impaired elderly and was revised in 2006. The aims of the LTC insurance system are to allocate limited resources to impaired elderly in a way that adequately reflects need and to reduce the burden on caregivers [6]. Services are allocated based on the Government-Certified Index (GCI), which indicates the amount that can be spent on services for a particular patient with a given GCI stage, basically ranging from 0 (need only support) to 5. Once this procedure has been completed, a care management agency steps are taken to provide the level of services indicated by the GCI stage. Disabled elderly can apply for care services including home help, nurse visits, day services, respite care, and institutional care (nursing homes, etc.). The GCI stage is reevaluated regularly twice a year or if necessary.

However, some researchers have claimed that the LTC insurance system does not adequately take into account problems associated with dementia, specifically Alzheimer's disease (AD) [7–9]. Patients with AD were supposed to require caring because of not only physical reasons but also other many reasons. Moreover, there have been few follow-up studies involving the GCI stage with requisite costs for the LTC insurance system for the demented elderly. Furthermore, as far as we know, there have been no studies investigating such transition in the GCI stage and requisite costs specifically related to the causes of dementia. The burden on caregivers for demented elderly differs largely depending on the causes of dementia [10]. The different causes of dementia may generate different costs [11].

In order to evaluate the extent of care services required by demented elderly, we need to ascertain the correlation between the GCI stage and requisite costs related to specific causes of dementia. The aim of this study is (1) to understand the present situation of LTC insurance costs among community-dwelling demented elderly through the 8-year followup of the GCI stage and requisite costs and (2) to ascertain whether LTC insurance costs can differ according to the causes of dementia.

## 2. Methods

Written informed consent was obtained after a complete description of the study had been given to all subjects or their informants. This study was approved by the Ethics Committee of Jikei University. 

### 2.1. Baseline Assessment

An epidemiological survey of dementia was conducted in Itoigawa city, a rural area of Japan, in 1998-1999. In 1998, there were a total of 33,120 inhabitants and 7,847 of them were over 65 years old. Two-phased, semistructured screening tests were conducted, including items on education, occupation, medical history, risk factors such as hypertension, diabetes, and hyperlipidemia, psychiatric and behavioral symptoms, severity of dementia measured by clinical dementia rating (CDR) [12], cognitive functions measured by mini-mental state Examination (MMSE) [13], and activity of daily living (ADL) measured by N-ADL [14]. N-ADL is an assessment tool for elderly ADL developed in Japan; it contains 5 domains including gait, daily milieu, clothing, eating, and excretion. Each domain scores from 0 to 10, with total score from 0 to 50. Zero shows that the subject requires complete assistance in every aspect, 50 means that the subject is independent.

Subjects who met the criteria (scored ≦19 on MMSE scored ≧20 on MMSE but had any psychiatric symptoms had been diagnosed to have dementia before the survey, etc.) in the first phase were recruited into the second phase. The second phase was conducted with a total of 1114 subjects, using a door-to-door survey by a clinical team including a psychiatrist and a public health nurse. A total of 271 of these subjects were CDR ≧1, 252 of them were CDR = 0.5 and 306 were CDR = 0. The other 285 subjects did not participate in the second phase due to refusal, death, or as a result of moving to other communities. The details of the design and methods of sampling in the baseline survey have been described elsewhere [15, 16].

A total of 271 of these subjects were diagnosed as having dementia. The diagnosis of dementia was established according to the DSM-IV criteria and Consortium to Establish a Registry for Alzheimer's Disease (CERAD) criteria [17, 18]. The demented subjects were classified into subgroups, according to the cause of dementia, on the basis of the DSM-IV criteria. 

### 2.2. Follow-Up Assessment

Among the 271 demented elderly, seven subjects moved to other communities (due mainly to institutionalization) or refused to participate in the follow-up investigation, and 55 subjects (or their family) did not enroll in the LTC insurance system. Two hundred and nine subjects were included in this study. We confirmed the age, sex, MMSE score, CDR score, causes of dementia, and any coexisting physical diseases of each of the 209 demented subjects.

After the LTC insurance system was launched in April 2000, we tracked the GCI stage and payment amounts from the government every month through December 2007, according to the data provided by the Health Improvement Section of the Welfare and Health Division of Itoigawa city. In addition, we compiled information pertaining to mortality (i.e., the date of death and causes of death), according to the data provided by the same agency.

### 2.3. Statistical Analysis

All data analyses were carried out using the SPSS16.0-PC software package. The significance of the differences between the groups was assessed by *t*-test for age, MMSE score, N-ADL score, and the caring costs; by Chi-square test with Fisher's exact test (extended) for sex, CDR, proportion of subjects, proportion of coexisting physical diseases; and by Mann-Whitney *U* test for caring periods. Median survival times were estimated based on the Kaplan-Meier method and survival curves were generated. Log-rank tests were performed to test the differences between the survival curves. A *P* value <0.05 (two tailed) was considered statistically significant.

## 3. Result

### 3.1. Demographic Background of 209 Subjects

A total of 209 demented elderly were certified to receive care insurance. The mean age in April 2000 was 84.5 ± 7.1. The sex ratio was 57 : 152 (male/female, female 73%), the CDR ratio was 78 : 72 : 59 (1 : 2 : 3), and the mean MMSE score was 15.7 ± 5.1. The mean N-ADL score was 31.4 ± 13.2. The most common cause of dementia was AD (*N* = 110, 52.6%), followed by vascular dementia (VaD) (*N* = 48, 23.0%), dementia with Lewy bodies, frontotemporal dementia, and other causes of dementia.

One hundred fifty-nine of the subjects suffered from other coexisting physical diseases in April 2000, such as hypertension (*N* = 77, 36.8%), brain disease (*N* = 41, 19.6%), heart disease (*N* = 36, 17.2%), digestive organ disease (*N* = 33, 15.8%), diabetes (*N* = 22, 10.5%), urologic disease (*N* = 14, 6.7%), hyperlipidemia (*N* = 14, 6.7%), and other diseases. 

At the end of our research period (December 2007), only 49 (23.4%) were alive. The average age at death was 89.0  ±  6.9; the most common cause of death was respiratory disease (*N* = 70), followed by heart disease (*N* = 34), brain infarction (*N* = 27), and other causes of death. The estimated median survival was 4.3 years (95% confidence interval, 3.6 to 5.1 years) from the beginning of the research period and 5.3 years from the diagnosis of dementia.

 Average duration of the certified to receive care insurance was 52.3 ± 32.1 months. 26.3% of the subjects required more than 84 months (7 years), and 14.8% of them required less than 12 months (1 year). Average costs (total payment amount from the government) was 7.39 ± 7.49 millionJapanese yen (JPY) (0~28.95 million JPY). Thirteen of them (6.2%) did not receive any payment although they were certified to receive care insurance benefit. Five of them suffered from AD, 3 suffered from VaD, and 5 suffered from other causes of dementia. At their initial certification in April 2000, two were GCI = 0 (need only support), two were GCI = 1, one was GCI = 2, two were GCI = 3, one was GCI = 4, and 5 were GCI5. After one year of follow up in April 2001, none of them applied for certification again. A comparison of the 13 subjects who received no payment and the remaining 196 subjects is shown in Table 1. Significant differences were found for sex (*P* = 0.004) and CDR grade (*P* = 0.044).

### 3.2. Difference between Alzheimer's Disease and Vascular Dementia

We also compared the GCI stage and requisite costs according to the causes of dementia. We excluded AD with cerebrovascular disease, dementia with Lewy bodies, frontotemporal dementia, and other causes of dementia because the number of these patients was too small to perform efficient statistical analysis. Finally, patients with AD and VaD were included in the analysis. For the care-giving costs analysis, we also excluded the above mentioned 13 subjects. This is because they did not receive any payment to compare and none of them applied for certification again after 1 year of follow up. Demographic variables of the two patient groups are summarized in Table 2. There were significant differences in age, sex, and proportion of coexisting physical diseases between the two groups. However, there were no significant differences in the CDR grade, with MMSE score at 2000. There were also no significant differences in the total N-ADL score and between each of 5 domains in N-ADL subscale between AD and VaD groups. Total duration of certified to receive care insurance until 2007 was 50.9 ± 31.6 month in AD patients, whereas 57.3 ± 30.3 month in VaD patients. There was no significant difference between the two groups (*P* = 0.260 by Mann-Whitney *U* test).

**Figure 1:**
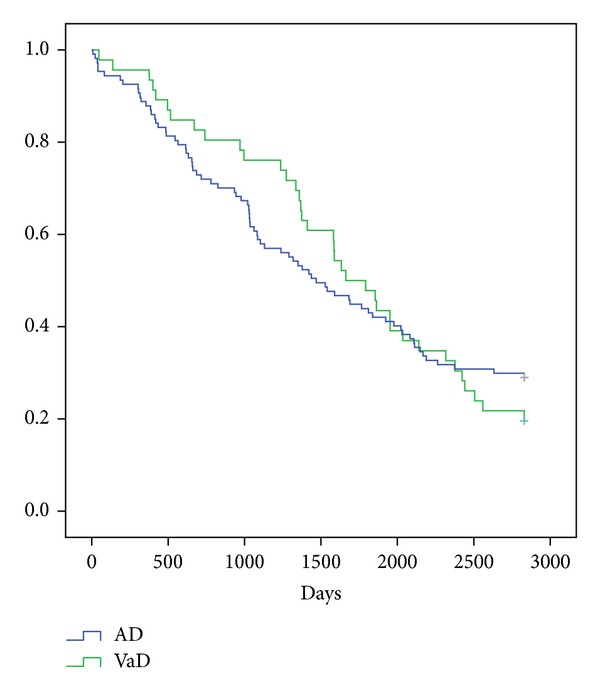
Survival curves of the two patient groups. AD: Alzheimer's disease; VaD: vascular dementia. No significant difference (*P* = 0.873) between the mortality rates of the two groups.

The survival curves of the two patient groups are shown in Figure 1. There was no significant difference (*P* = 0.873) between the mortality rates of the two groups. 

**Figure 2:**
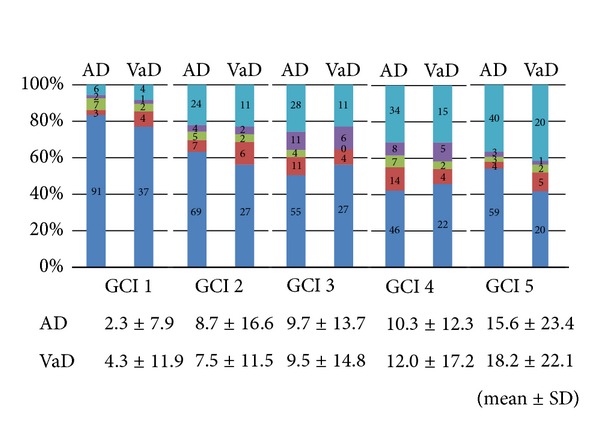
Percentages for each time period (0–3 months/4–6 months/7–9 months/10–12 months/over 13 months) that AD and VaD patients spent at each GCI stage. Mean duration for each GCI stage (month). AD: Alzheimer's disease; VaD: vascular dementia. Number in the figure is the number of the patients in each time period. As the GCI stage increased, patients in both groups spent a longer duration in each of the subsequent periods. However, there was no significant difference between the two groups in the duration of any of the GCI stages.

GCI stage and payment amounts generally increase as time passes in both patient groups; however, the patterns of change vary widely according to each individual. Therefore, we investigated the total duration of time and the total payment amounts recorded for each GCI stage.  Figure 2 shows percentages for each time period (0–3 months/4–6 months/7–9 months/10–12 months/over 13 months) that patients spent at each GCI stage. One AD subject died soon after the begining of the research period. Therefore, we excluded this subject from Figure 2. Generally, patients in both groups spent a longer duration in each of the subsequent periods as the GCI stage increased. However, there was no significant difference between the two groups in the duration of any of the GCI stages (by Mann-Whitney *U* test).

AD patients required an average of 6.99  ±  6.85 million JPY, and VaD patients required an average of 9.97 ± 8.62 million JPY. Average payments per month for each GCI stage for both AD and VaD groups are summarized in Table 3. 

There was a significant difference between the two groups in the total payment amount per total certified period (*P* = 0.047). There was also significant difference between the two groups in GCI 5 (*P* = 0.037); VaD patients required a higher costs than AD groups.

## 4. Discussion

This study is the first to practice the follow-up study of the GCI stage and requisite costs for the LTC insurance system among community-dwelling demented elderly according to the causes of dementia. During 8 years of followup since the LTC insurance system was launched, 77% of the demented elderly died, and the average age at death was 89.0 years old. The median survival was 5.3 years from the diagnosis of dementia. Previous studies reported the median survival time from the diagnosis of dementia as 5.7 years [19] and 3.3 years [5]; our result does not differ largely from these results.

The average LTC cost (total payment amount from the government) was 7.39 million JPY (0~28.96 million JPY), or 1.72 million JPY per year. This result is based on the LTC cost of single local government (Itoigawa city). There is a possibility that LTC costs differ between each local government according to its financial state. We need further reports based on other local governments in order to generalize our result. However, there are some previous researches considering care costs in other countries. Recent research in Germany mentioned that care costs for dementia patients in community were average 47,747 (Euros) per patient annually, or 4.91 million JPY (1 Euro = 103.3 JPY on September 24, 2011) [20]. Eighty percent of that total costs were for informal care, 9396 (0.97 million JPY) were for formal care. Even though the LTC systems of the two countries (i.e., the way to decide the payment amount and the way to pay for care giving) were different, and the demographic background of two researches (i.e., all kinds of dementia were included in Germany subjects) was different, the results of our study in Japan and the result in Germany do not differ largely when changes in exchange rates have been taken into consideration. And therefore, we believe that our results are valid enough. 

Six percent of the demented elderly patients (primarily males and severely demented subjects) did not receive any payment although they were eligible. Furthermore, none of them applied for certification again after 1 year of followup. The reason why these subjects did not receive benefits is still unclear; this may have been due to their own refusal or some other familial reasons. We should be careful not to make these subjects being left out of the support.

In our research, there was no significant difference between the mortality rates of AD and VaD patients. This result is consistent with previous studies [5, 19] reporting the mortality rates of AD and other demented patients. However, in our research, VaD patients received significant higher total payments than AD patients. Furthermore, VaD patients required significant higher costs per total certified period than AD patients especially in GCI 5 stage. VaD patients required higher caring cost in the high CGI stage, although there was no significant difference in the duration of the GCI subsequent periods. 

There are a few possible reasons of this higher cost of VaD patients. First, the incidence of coexisting physical diseases is higher in VaD patients than AD patients (92% versus 64%); although VaD patients were younger, and the total and each of subscales (gait, daily milieu, clothing, eating, and excretion) of N-ADL scores did not differ between the two groups among the background factors in 2000. These coexisting physical diseases include vascular risk factors such as hypertension, diabetes, and hyperlipidemia. VaD patients generally have more risk factors such as diabetes or hypertension [21]. These risk factors may cause other coexisting physical diseases such as brain disease and heart disease. During the 8 years of the follow-up period, numbers and the severity of these coexisting physical diseases may had been exacerbated and ADL levels of VaD patients may had been more exacerbated, and resulting from higher caring cost. Even though demented elderly require caring for many reasons, VaD patients may require higher costs due to their physical conditions. However, we could not follow the rate of coexisting physical diseases and transition of ADL during the follow-up period. There is also significant difference in sex ratio; there are relatively more male subjects in VaD groups. However, there is a research in Germany that females incurred greater LTC costs than males of the same age [22]. Therefore, we believe that there is little possibility that this sex ratio may have an effect on the caring costs. The aims of this study are to understand the present situation; therefore, we dare not adjust these background factors.

Second, there is a possibility that the kind of provided service (home help, nurse visits, day services, respite care, and institutional care) differs between the two groups. There may be more institutionalized VaD subjects in GCI 5 who required higher caring cost. However, we followed only GCI stage and payment amounts in this study and we cannot investigate the kind of provided service. Therefore, we cannot certify this hypothesis.

 There are some researches comparing healthcare costs of VaD and other diseases in the United States [22, 23]. They reported that healthcare costs for VaD patients were substantially higher than other groups because of higher hospital costs. They also reported that the pattern of healthcare utilization for VaD was substantially different from other groups; VaD patients had lower utilization for physician office visits and prescription drugs. VaD patients may not receive adequate medical care, placing them at greater risks for more costly inpatient care. Although healthcare costs and LTC insurance costs are different and the systems in Japan and United States are different, their assumption can be applied to our results.

There are a few methodological issues that should be taken into consideration to appreciate our results fully. First, we compared only patients with AD and VaD because the number of other causes of dementia is too small to make an effective analysis in this research. In our cohort, there were few patients with DLB and FTLD. This may be because our basic research was conducted in 1998-1999 when the concept of DLB was not so widely known at that time. However, DLB and FTLD were assumed to require higher costs because of their behavioral symptoms and neurological symptoms. Further research on other causes of dementia is required.

Second, in this research, we focused on the causes of dementia and tracked only the GCI stage and payment amounts as outcome measurements. It is difficult for us to discuss the influences of other factors such as residential state, numbers of family caregivers, new physical disease that could have occurred during the follow-up period, the transition of ADL of subject, and the kind of provided service.

Third, out data is based only on the costs for LTC insurance system; informal care-giving costs, and other medical costs are not included in this research. We cannot discuss the total care-giving costs and caregiver's burden in this study. We need further researches on the total care costs of demented elderly. 

 In conclusion, our results indicate that the requisite costs of LTC insurance system for demented elderly differ depending on the causes of dementia. In order to reduce costs for LTC insurance of demented elderly, prior control of the risk factors may be important to prevent suffering from VaD. Further research on the detailed effects of cognitive function and behavioral problems for care-giving costs for demented elderly is required.

## Figures and Tables

**Table 1:** Comparison between subjects who received payment and those who did not.

	No payment	Payment	*P* value
	(*N* = 13)	(*N* = 196)	
Mean age (at 2000)	86.8 ± 8.4	84.4 ± 7.0	0.233 (*t* test)
Sex (M : F)	8 : 5	49 : 147	0.004 (*χ* ^2^ test)
CDR grade (1 : 2 : 3 at 2000)	1 : 8 : 4	77 : 64 : 55	0.044 (*χ* ^2^ test)
MMSE score (at 2000)	16.7 ± 6.6	15.7 ± 5.0	0.601 (*t* test)
N-ADL score (at 2000)	25.5 ± 15.1	31.8 ± 13.0	0.141 (*t* test)

**Table 2:** Demographic variables of AD and VaD patients.

	AD (*N* = 110)	VaD (*N* = 48)	*P* value
Mean age (at 2000)	85.8 ± 7.0	82.2 ± 5.9	0.002 (*t* test)
Sex (M : F)	15 : 95	15 : 33	0 .009 (*χ* ^2^ test)
CDR grade (1 : 2 : 3 at 2000)	35 : 40 : 35	23 : 15 : 10	0.134 (*χ* ^2^ test)
MMSE score (at 2000)	15.7 ± 5.0	14.8 ± 5.5	0.413 (*t* test)
N-ADL score (at 2000)	32.7 ± 12.3	28.6 ± 14.5	0.124 (*t* test)
Coexisting physical diseases (any/nothing at 2000) (%)	71 : 39	44 : 4	0.000 (*χ* ^2^ test)
	64%	92%	
Survival ratio (alive/dead at 2007)	39 : 79	9 : 31	0.210 (*χ* ^2^ test)
Total duration of certified until 2007 (month)	50.9 ± 31.6	57.3 ± 30.3	0.260 (Mann-Whitney *U* test)

AD: Alzheimer's disease; VaD: vascular dementia.

**Table 3:** Average payments for each GCI stage for both AD and VaD groups per month.

	AD (*N* = 105)	VaD (*N* = 45)	*P* value
GCI 1	7.2 ± 27.4	16.7 ± 48.3	0.129
GCI 2	34.3 ± 64.4	43.4 ± 69.6	0.436
GCI 3	67.1 ± 83.6	67.6 ± 91.7	0.969
GCI 4	103.7 ± 105.7	121.2 ± 124.2	0.379
GCI 5	94.7 ± 118.5	140.8 ± 113.6	0.037

Total	113.4 ± 76.8	141.1 ± 97.9	0.047

(thousand JPY/month).

AD: Alzheimer's disease; VaD: vascular dementia.

Mean duration of both patient groups for each GCI stage was summarized in Figure 2.
